# Targeting oestrogen to kill the cancer but not the patient

**DOI:** 10.1038/sj.bjc.6601627

**Published:** 2004-03-02

**Authors:** J S Lewis, D Cheng, V C Jordan

**Affiliations:** 1 Robert H Lurie Comprehensive Cancer Center, Feinberg School of Medicine, Northwestern University, 303 East Chicago Avenue, Olson Pavilion, Room 8258, Chicago, IL, 60611, USA

**Keywords:** tamoxifen, raloxifene, aromatase inhibitor, estrogen receptor, apoptosis

## Abstract

The link between sex steroids and the development and growth of breast cancer has proved to be an invaluable clue for advances in the prevention and treatment of breast cancer. The identification of the oestrogen receptor (ER) not only allowed advances in the molecular endocrinology of oestrogen action, but also provided a target for antioestrogenic therapeutic agents. However, the application of long-term or indefinite treatment regimens has consequences for the breast cancer. New forms of resistance, based upon enhanced cellular survival networks independent of ER and the suppression of apoptotic mechanisms, develop and then evolve. Remarkably, low concentrations of oestrogen collapse survival pathways and induce apoptosis in completely antihormonally refractory breast cancer. However, recurrent oestrogen-stimulated disease is again sensitive to antihormonal therapy. The novel reapplication of the ER as a therapeutic target for apoptosis is emerging as a new strategy for the long-term targeted maintenance treatment of breast cancer, and in formulating a targeted strategy for endocrine independent cancer.

There is an expanding clinical database that implicates oestrogen and progestins (hormone replacement therapy, HRT) in the development and growth of breast cancer. Evidence to support this conclusion comes from two major clinical sources: clinical studies of HRT, initially designed to determine the benefits of replacement approaches on postmenopausal women's health (Writing Group for the Women's Health Initiative Investigators, 2002; Million Women Study Collaborators, 2003) and the successful clinical strategy of treating breast cancer by blocking oestrogen action.

The Million Woman Study provides powerful (Million Women Study Collaborators, 2003) information about the actual impact of HRT on the incidence of breast cancer. A total of 1 084 110 UK women aged 50–64 years were recruited to determine the association of HRT use with breast cancer incidence and death. It is estimated that 10 years use of HRT produces 19 additional breast cancers per 1000 users. These data extrapolate to an estimated total of an excess of 15 000 breast cancers associated with HRT over the past decade in the UK.

In contrast to the effects of hormone replacement on the incidence of breast cancer, the use of either an antioestrogen, tamoxifen, to block the action of oestrogen in breast cancer (EBCTCG, 1998), or an aromatase inhibitor, to prevent oestrogen synthesis in postmenopausal patients (ATAC Trialist Group, 2002), is effective, and is considered to be the standard treatment strategy for breast cancer. Indeed, the concept of antioestrogenic interventions has been advanced with the reduction of risk from breast cancer by using tamoxifen (Cuzick *et al*, 2003) or raloxifene (Cummings *et al*, 1999), as well as suggestions for the evaluation of a number of aromatase inhibitors as chemopreventives in high-risk postmenopausal populations.

To the casual observer, the clinical evidence appears to demonstrate that oestrogen is detrimental to women's health and especially implicated in breast cancer development and growth. However, there have been consequences to current antihormonal strategies and we will propose new concepts about antihormonal drug resistance in breast cancer, which can be rapidly incorporated into the treatment plan. In otherwords, there are now opportunities to kill sensitised tumour cells with oestrogen and advance a new innovation in therapeutics.

## CLASSICAL CONCEPT OF TUMOUR TARGETING

The discovery of the ER as a critical component of the oestrogen signal-transduction pathway in target tissues (Jensen and Jacobson, 1962) and the utilisation of this knowledge as a target for antihormonal therapy in breast cancer (Jensen and Jordan, 2003) improved the survival prospects for millions of breast cancer patients. However, the advance with antioestrogens occurred not only because of targeting the ER, to prevent oestrogen-stimulated tumour growth, but also because of the application of the appropriate duration of treatment. During the 1970s, when tamoxifen was initially being evaluated as an adjuvant therapy in patients, laboratory studies demonstrated that longer durations of complete antihormonal therapy were likely to provide more benefit for patients than shorter durations of therapy (Lerner and Jordan, 1990). At the time, the majority of clinical trials elected to use 1 year of therapy, because there was a sincere concern that longer durations would enhance the possibility of premature drug resistance. Following the methodical evaluation of randomised clinical trials, conducted over the past 20 years, it is now clear that 1 year of adjuvant tamoxifen is only minimally effective, but 5 years of tamoxifen produces an increase in disease-free and overall survival (EBCTCG, 1998). The use of long-term (5 years) adjuvant antihormone therapy is now standard for the treatment of breast cancer therapy.

Attempts to enhance the effectiveness of adjuvant tamoxifen by increasing the duration of treatment from 5 to 10 years have been disappointing (Fisher *et al*, 2001). It appears there is a reduced effectiveness of the antitumour actions of tamoxifen, possibly because of developing drug resistance, but also an increase in oestrogenic side effects such as endometrial cancer and blood clots. In contrast, the use of a non-crossresistant aromatase inhibitor as a continuing adjuvant after 5 years of adjuvant tamoxifen reduces the incidence of recurrence and contralateral breast cancer by nearly 50% compared to no treatment (Goss *et al*, 2003). Thus, the cycling of antihormonal strategies can maintain patients disease free for long periods.

Clinical trials of adjuvant tamoxifen therapy also provide an invaluable insight into the appropriate duration of treatment necessary for the evaluation of the prevention of primary breast cancer. The Oxford Overview of clinical trials (EBCTCG, 1998) demonstrated that 1 and 2 years of adjuvant tamoxifen produce only modest decreases in contralateral breast cancers, but 5 years of adjuvant tamoxifen reduce contralateral breast cancer by 50%. These data are consistent with the findings of the NSABP P-1 trial that 5 years of tamoxifen reduce the incidence of invasive and noninvasive breast cancer in high-risk pre- and postmenopausal women, by approximately 50% (Fisher *et al*, 1998). Currently, in the United States, appropriately selected high-risk women can use a course of 5 years of tamoxifen to reduce their risk of breast cancer.

The treatment of breast cancer has changed dramatically during the past 15 years, with all patients now receiving long-term antihormonal therapies, whether it is tamoxifen (EBCTCG, 1998), aromatase inhibitors (ATAC Trialists' Group, 2002), or LHRH superagonists plus tamoxifen (Emens and Davidson, 2003). However, the intense laboratory study of nonsteroidal antioestrogens led to the recognition of selective oestrogen receptor modulation (SERM) (Jordan, 2001), and the idea that SERMs could be used to treat and prevent osteoporosis but prevent breast cancer as a beneficial side effect (Lerner and Jordan, 1990). Raloxifene, a molecule related to tamoxifen, is used to treat and prevent osteoporosis with a reduction of breast cancer (Cummings *et al*, 1999). In the wake of the controversy surrounding the negative effects of HRT, there are new opportunities to develop novel SERMs to address the prevention of osteoporosis, coronary heart disease (CHD) and breast cancer. Raloxifene is currently being evaluated for the prevention of breast cancer and CHD in high-risk postmenopausal women, so it could become the first multifunctional medicine.

Despite the fact that SERMs introduce a new dimension into therapeutics for the prevention of osteoporosis and CHD, the question of unlimited treatment durations with SERMs will have consequences for the natural history of breast cancer. Early reductions in the incidence of ER-positive disease (Fisher *et al*, 1998; Cummings *et al*, 1999) will potentially result in SERM-resistant breast cancers, thereby confronting the oncologist with unanticipated and complex treatment decisions in an increasing proportion of women. However, laboratory studies have established a new understanding of SERM resistance that could potentially convert breast cancer from an acute to a chronic, controllable disease.

### CHANGES IN THE UNDERSTANDING OF ANTIHORMONE RESISTANCE

**Figure 1:**
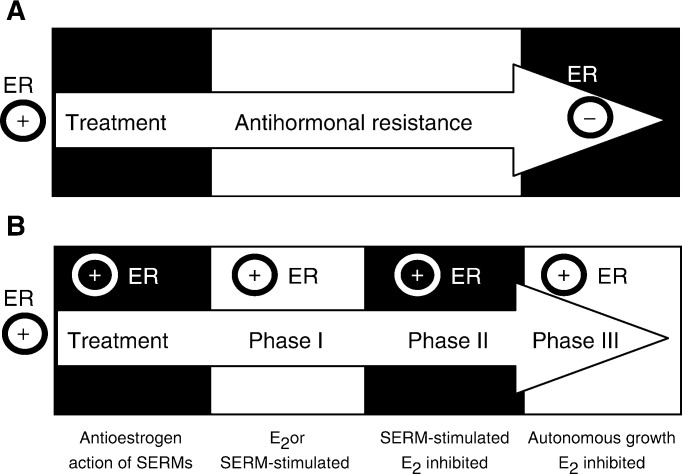
The evolution of antihormonal resistance. (**A**) About 20 years ago, it was believed that oestrogen receptor-positive (ER+) tumours would usually be expected to respond to oestrogen withdrawal or a selective oestrogen receptor modulator (SERM) such as tamoxifen, but eventually resistance would occur because ER− cells would overgrow the tumour. (**B**) Emerging laboratory and clinical evidence suggests that SERM resistance evolves from acquired resistance (Phase I) to Phase II, where any SERM will maintain growth, whereas, unliganded ER does not provoke growth. However, oestrogen at physiological levels causes rapid apoptosis. In Phase III, tumours are completely resistant to all antihormonal therapies and grow spontaneously. Nevertheless, physiological concentrations of oestrogen causes rapid apoptosis.

During the past 20 years, there has been an important change in the understanding of drug resistance to the antioestrogen tamoxifen. In the early 1980s, tamoxifen was anticipated to be effective in ER-positive breast cancers, but ineffective in ER-negative disease. Resistance to tamoxifen would develop because the ER-positive tumour cells would be controlled, but eventually these would be overwhelmed by the outgrowth of ER-negative breast cancer cells (Figure 1A

). However, this model was not consistent with the known clinical observation that select patients with metastatic breast cancer could be maintained on successive endocrine therapies for prolonged periods.

It is now clear that there are two types of drug resistance to tamoxifen: intrinsic resistance, where the tumour is either ER negative or ER positive with pre-existing enhanced survival pathways (HER2/neu plus a coactivator molecule AIB1) (Osborne *et al*, 2003), and acquired resistance where an ER-positive tumour that initially responds to treatment now becomes tamoxifen-stimulated and grows in response to either tamoxifen or oestrogen. An ER-positive breast tumour that initially responds to an SERM eventually develops acquired resistance by clonal selection for increased cell surface signalling that subverts the SERM ER complex through enhanced phosphorylation cascades. The laboratory description of acquired resistance forms the scientific basis for the understanding of current therapeutic interventions with aromatase inhibitors or fulvestrant as second-line agents, following the development of clinical resistance to tamoxifen. Aromatase inhibitors block oestrogen synthesis and fulvestrant destroys the ER to prevent the growth of breast cancers with acquired tamoxifen resistance. However, despite the remarkable investment in a broad range of antihormonal therapies, the actual advance in improved survival and reduced side effects has been modest.

The successful control of breast cancer with antihormonal therapy requires years of successive treatments. An obstacle to the progress in therapeutics is a clear understanding of the changes that occur in the breast cancer cell, as a consequence of exhaustive antihormonal therapies. It is presumed that the cancer cell must create a sophisticated survival network and suppress the natural process of apoptosis to subvert the continuous inhibitory signal through the ER. Until recently, no laboratory models replicated the former clinical scenario, but the deficiencies in our knowledge are being corrected.

Drug resistance to tamoxifen evolves through three phases: Phases I and II both require tamoxifen, or a related SERM (O'Regan *et al*, 2002), to maintain growth, but in Phase III the ER-positive tumour is refractory to all antihormonal therapies and growth is SERM independent (Jordan *et al*, 2003) (Figure 1B).

Remarkably, the response of the breast tumour to oestrogen during the evolution of drug-resistance changes dramatically, from initially being growth stimulatory to becoming completely inhibitory after 5 years of antihormonal therapy. Apoptosis is initiated in response to minute concentrations of oestradiol, which results in dramatic tumour regression in heterotransplanted athymic mice. When this phenomenon was first noted and reported in the early 1990s (Wolf and Jordan, 1993), it was suggested that a woman's own oestrogen actually destroyed the micrometastases that were presensitised by 5 years of tamoxifen treatment. In otherwords, stopping tamoxifen at a critical time (5 years) was responsible for the long-term survival statistics. While the concept may have some veracity, the knowledge that minute concentrations of oestrogen can have a dramatic effect on tumour cell death could have important clinical implications for treatment. Indeed, a clear understanding of the mechanism of Phase II and III resistance is becoming increasingly important for the treatment of metastatic breast cancer.

The current focus on exhaustive antiendocrine therapy may in fact be disadvantageous for patients. The antitumour action of oestrogen in Phase II tamoxifen resistance can paradoxically be reversed by fulvestrant (Osipo *et al*, 2003). In otherwords, oestradiol causes rapid tumour regression, fulvestrant causes tumourstasis, but a combination of oestradiol and fulvestrant causes robust tumour growth. If Phase II or III drug resistance to tamoxifen can be shown to occur in patients, then an oestrogen-rich environment would subvert the actions of fulvestrant. Obviously, one could enhance the probability of a response to fulvestrant by administering an aromatase inhibitor, but perhaps oestrogen should be pursued as a targeted alternative. To advance this idea, it would be valuable to examine whether the observations in the laboratory with the evolution of tamoxifen drug resistance apply to other SERMs and to breast cancer cells that have adapted to oestrogen deprivation, that is, as an expression of resistance to aromatase inhibitors.

## EXPANSION OF THE CONCEPT OF OESTROGEN-INDUCED APOPTOSIS

**Figure 2:**
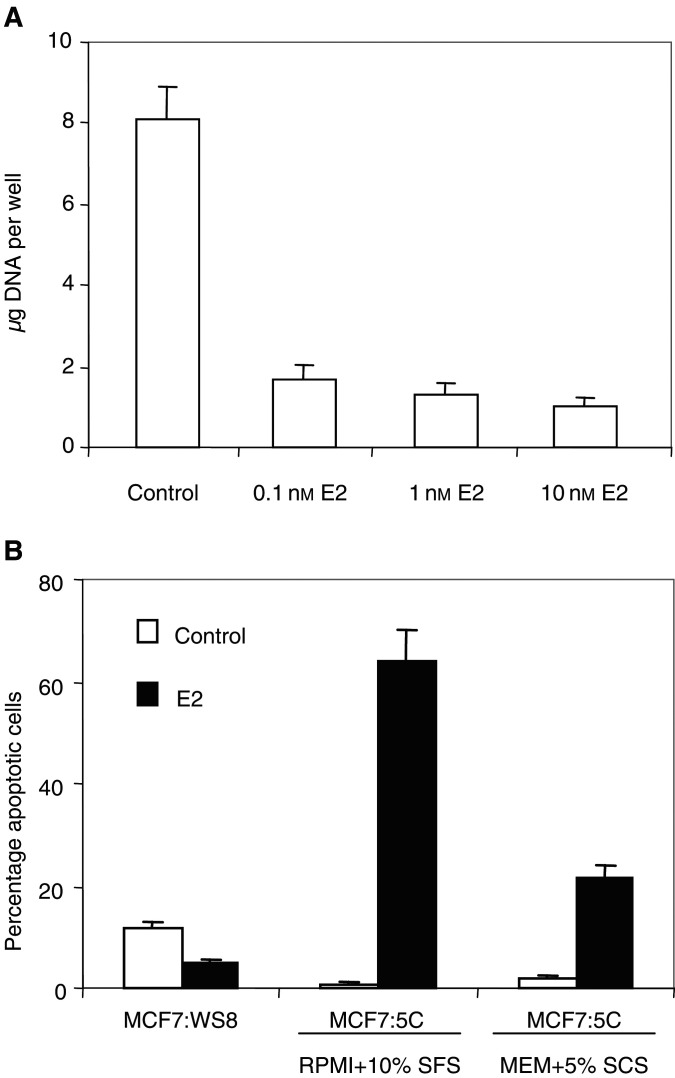
Oestradiol inhibits the growth of MCF-7:5C cells and induces apoptosis. MCF-7:5C cells were cloned from wild-type MCF-7:WS8 cells following long-term growth (∼1 year) in oestrogen-free RPMI medium containing 10% (v v^−1^) dextran charcoal-stripped (DCC) foetal bovine serum (FBS), 2 mM glutamine, 100 U ml^−1^ penicillin-streptomycin, 6 ng ml^−1^ bovine insulin, and 1 × nonessential amino acids. (**A**) For DNA assays, MCF-7:5C cells were seeded into 12-well plates at a density of ∼20 000 cells per well in RPMI medium. The cells were left for 24 h to acclimatise, and then treated with 0.1, 1, or 10 nM oestradiol (E_2_) for a total of 6 days, with the control cells receiving <0.1% ethanol vehicle. Cells were re-fed on days 3 and 5. Total DNA (*μ*g) per well was used to measure cell growth. The data represent the average of five separate experiments. (**B**) Apoptotic cells were identified/quantified by double staining with recombinant FITC-conjugated annexin V and propidium iodide (PI), using the Annexin V-FITC kit (Immunotech, Beckman Coulter). For experiments, MCF-7:WS8 and MCF-7:5C cells were seeded in 100 mm plates at a density of 1 × 10^6^ per plate in either oestrogen-free RPMI medium containing 10% DCC stripped fetal bovine serum (SFS) or MEM containing 5% DCC stripped calf serum (SCS). The cells were left for 24 h to acclimatise and then treated with either 1 nM E_2_ or less than 0.1% ethanol vehicle (control) for 72 h. Data shown represent three separate experiments. It should be noted that oestradiol treatment of MCF-7:WS8 cells in oestrogen-free MEM media containing 5% SCS did not have any significant effect on apoptosis (data not shown).

Song *et al* (2001) were the first to demonstrate that oestradiol causes apoptosis in breast cancer cells that have been adapted to grow in an oestrogen-free environment for prolonged periods. These investigators suggested that their data provided an explanation for the effectiveness of high-dose diethylstilboestrol formally used to treat breast cancer in elderly postmenopausal women, that is, women who were 10–25 years after menopause. Thus, a switch occurs in the cancer cell from oestrogen stimulating growth to oestrogen causing cell death after prolonged oestrogen deprivation. However, and perhaps more importantly, the apoptotic effect occurs with low concentrations of oestradiol. We illustrate the principle in Figure 2B

by comparing and contrasting the action of oestradiol in wild-type breast cancer cells and an ER-positive progesterone receptor-negative, oestrogen-deprived clone referred to as MCF-7 5C (Jiang *et al*, 1992). Of interest is the observation that alterations in the media can enhance the actions of oestradiol as an apoptotic agent. Clearly, this is an area of research activity worthy of pursuit as the proposed therapeutic use of long-term aromatase inhibition can potentially reconfigure the cellular response to oestrogen.

The other relevant clinical scenario is the response of occult breast cancer cells to indefinite raloxifene administration for the treatment and prevention of osteoporosis. Long-term culture of breast cancer cells in 1 *μ*M raloxifene results in adaptation of cell-survival mechanisms and raloxifene resistance. Transplantation of cells into athymic mice demonstrates Phase II SERM resistance; tamoxifen or raloxifene stimulates growth, no treatment (no ER binding ligand) results in no growth, but oestradiol causes apoptosis and rapid tumour regression (Liu *et al*, 2003). Thus, a general principle is emerging that merits investigation in the laboratory to discover mechanisms that could be targeted and amplified.

## MOLECULAR MECHANISMS

**Figure 3:**
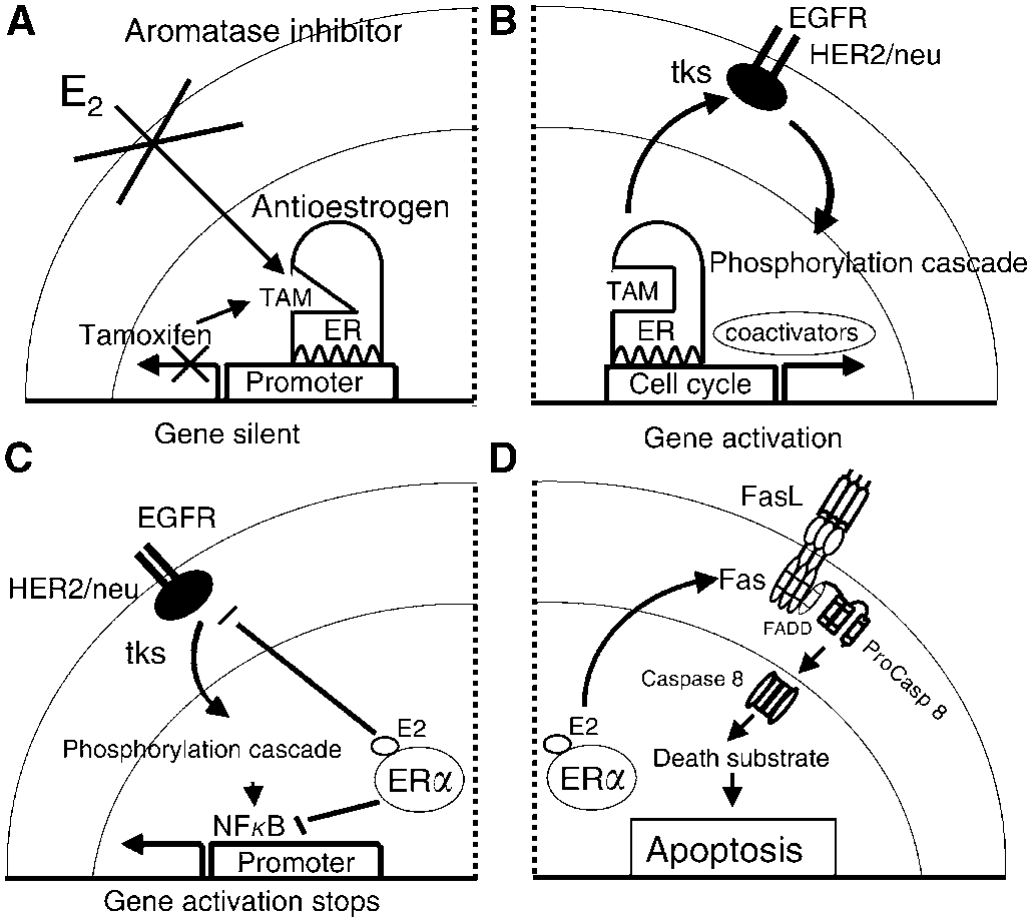
The development of tamoxifen (TAM)-resistant breast cancer and the changing role of oestradiol (E_2_) in the life and death of ER-positive cancer cells. (**A**) E_2_-stimulated growth is inhibited by the use of an aromatase inhibitor to block oestrogen synthesis or TAM to block the ER and prevent oestrogen-stimulated gene transcription. Optimal antioestrogenic effects occur in the absence of pre-existing cell surface signalling mechanisms. (**B**) Prolonged use of TAM promotes an increase in HER2/neu cell surface signalling that creates a survival pathway phosphorylating the TAM ER complex and coactivator proteins. The transcription complex becomes activated to enhance gene activation and TAM-stimulated growth. If this is Phase I resistance, then oestrogen will also promote growth; so an aromatase inhibitor is an appropriate second-line therapy. If it is Phase II resistance, E_2_ causes apoptosis. (**C**) In Phase II tamoxifen resistance, the E_2_ ER complex collapses the survival mechanisms by dramatically reducing the level of cell surface signalling by preventing HER2/neu mRNA transcription and the nuclear level of NF*κ*B, a transcription factor. (**D**) In Phase II tamoxifen resistance, the E_2_ ER complex also enhances the synthesis of Fas receptor mRNA and protein which, in the presence of Fas ligand (FasL) activates caspase 8 and a cascade of events resulting in apoptosis. These figures are summaries of the mechanisms described in Osipo *et al* (2003) and Liu *et al* (2003).

The target site-specific actions of SERMs are not well understood, but there is an emerging understanding of the modulation of the SERM ER complex in breast cancer (Figure 3

). Antioestrogenic action appears to be dominant in a cancer cell with no cell surface signalling, and the SERM ER complex binds preferentially to corepressor molecules to prevent gene activation and cell replication (Figure 3A). In contrast, the oestrogen-like actions of an SERM become dominant in acquired resistance when cells are selected with enhanced cell surface signalling, reduced corepressors but increased coactivator molecules (Figure 3B). This molecular mechanism could serve as a working model for Phase I drug resistance. It is plausible that the subsequent evolution of drug resistance into Phase II results from an enhanced sophistication in establishing survival pathways during continuing selection in an oestrogen-deprived environment. Ultimately, the ER appears to become redundant for growth in Phase III drug resistance (Jordan *et al*, 2003). However, it is the remarkable switch from oestrogen-stimulated growth in breast cancer to oestrogen-stimulated death that merits investigation. Song *et al* (2001) first focused attention on the fas, fas ligand pathway as a potential mechanism of oestrogen-induced apoptosis in oestrogen-deprived breast cancer cells. Study of molecular mechanisms for oestrogen-induced tumour regression have recently been extended with the demonstration that oestrogen simultaneously collapses survival mechanisms (HER2/neu NF*κ*B) in the Phase II SERM-resistant tumour, and enhances the expression of the fas receptor (Liu *et al*, 2003; Osipo *et al*, 2003) (Figure 3C, D). Clearly, much needs to be done to understand the new molecular endocrinology of oestrogen action, but the knowledge now creates opportunities for novel applications in therapeutics.

## CLINICAL CORRELATIONS

Current clinical practice is focused on the premise that oestrogen is the principal growth stimulator in select ER-positive breast cancers. The application of this principle over the past 30 years has been at the forefront of targeted therapeutics, and has undoubtedly saved lives. However, clinical clues are emerging that the practice of exhaustive antihormonal therapy is not always appropriate, and useful palliation can occur with an application of the obsolete modality of pharmacological oestrogen treatment (diethylstilboestrol, 5 mg three times a day). Renewed interest in re-treating endocrine refractory disease with high-dose oestrogen has demonstrated improvement in the anecdotal patient (Ingle, 2002) and remarkable responses in metastatic breast cancer patients treated exhaustively with antihormonal therapies (Lonning *et al*, 2001). Out of 32 evaluable patients, four had complete response, six had partial response and two had stable disease. These encouraging data require confirmation, before any change in medical practice can be recommended, but for the individual patient with completely refractory disease the potential for palliation is clear. More importantly, there is now a renewed conversation between the laboratory and the clinic that offers opportunities not only to enhance the duration of responses based on retargeting the ER, but also to improve the proportion of response rates, based on an enhanced understanding of the survival and death pathways that could potentially be manipulated.

## OPPORTUNITIES FOR TARGETING OESTROGEN TO THE TUMOUR

Professor Paul Erlich established the foundation for targeted therapeutics by describing the successful process for treating infectious disease. An effective treatment is based on selective toxicity to destroy the disease and not the patient. To achieve that goal, an appropriate laboratory model for the disease in question should be used to identify active synthetic molecules that will selectively destroy the disease *in vivo* under laboratory conditions. The successful chemotherapeutic candidate can subsequently be evaluated in clinical trials to establish ‘real world’ efficacy and selectivity. The process has been remarkably successful during the past Century, with the development of a diverse range of antibacterial agents and drugs to either prevent or palliate parasitic disorders. Unfortunately, the realisation of the goal of targeting cancer selectively has, until recent times, remained elusive. Not that there has not been a sincere attempt to achieve the goal. About 100 years ago, Professor Paul Erhlich was the first to apply the principles of chemotherapeutic drug discovery to cancer cures. His approach was not successful. He declared, ‘I have wasted 15 years of my live in experimental cancer research’, the year before he died on 20th August 1915.

What has become increasingly clear is that establishing target site specificity is not simple, especially in cancer, and that, for the application of the Erhlich method in general, there are consequences of launching an attack that is not complete – drug resistance. The success in targeting breast cancer initially developed slowly by the reinvention and retargeting of existing molecules that had not succeeded in their primary applications. Tamoxifen, discovered in a fertility-control program, was reinvented as a breast cancer drug and subsequently targeted to the ER (Jordan, 2003). Raloxifene, discovered in a breast cancer program and targeted to the ER, was reinvented as a preventive for osteoporosis with breast and uterine safety. After 30 years of clinical usage, the ubiquitous application of SERMs has now provided clues to progress by retargeting the ER with oestrogen in SERM-resistant disease. However, rather than returning to the therapeutic modality of the 1960s by reintroducing high-dose oestrogen therapy for a few select patients, the new knowledge emerging from the laboratory now creates novel scientific and clinical opportunities to target the ER and extend response rates, cycle antihormonal therapies, enhance response rates and determine the precise molecular mechanism of ER-mediated apoptosis, so the knowledge could potentially be exported to kill ER-negative tumour cells.

There are at least two dimensions to consider when applying the targeted action of oestrogen to the tumour: the nature of the oestrogen ER complex and the length of time that oestrogen must be administered to initiate the apoptotic cascade. The ER can bind an enormous range of ligands with diverse shapes and structures. To date, laboratory and clinical studies of ER-mediated apoptosis have only used either oestradiol or DES. However, recent studies of the molecular biology of oestrogen action have defined two classes of ER complex. This is because the shape of the ligand preprograms the actual external shape of the ER complex (Bentrem *et al*, 2003). The planar oestrogens, oestradiol, DES and the phyto-oestrogens genistein and coumestrol are class I oestrogens that rely on helix 12 in the ligand-binding domain to seal the oestrogen molecule into the hydrophobic pocket (Bentrem *et al*, 2003). This causes activating function (AF) 2 to be triggered, and synergise with AF-1 at the other end of the complex during the formation of a gene transcription complex. In contrast, a nonplanar oestrogen binds in the ligand binding domain, but prevents activation of AF-2. Helix 12 cannot seal the ligand in the hydrophobic pocket, and the transcription complex occurs through the triple site interaction of AF-1, D351 and select acidic amino acids in helix 12 (Bentrem *et al*, 2003). The comparative testing of a range of phytoestrogens and the active constituents of conjugated equine oestrogens, equilin and equilenin, for their activity as apoptotic agents in Phase II and III antihormone drug resistance, will establish which types of low-dose oestrogen could be used in clinical studies.

An important laboratory observation is that the apoptotic response of SERM-resistant breast cancer occurs, not with pharmacologic doses of oestrogen but physiologic oestradiol in the postmenopausal range (Yao *et al*, 2000). The majority of SERM-resistant tumours regress completely, but the few that regress but regrow are again completely responsive to tamoxifen that prevents oestradiol-stimulated growth. Although the precise nature of switching mechanisms from tamoxifen stimulated and oestrogen killing to oestrogen stimulated and tamoxifen blocking has not been established, recent studies (Liu *et al*, 2003; Osipo *et al*, 2003) show that oestradiol rapidly collapses the survival mechanisms (HER2/neu and NF*κ*B) that support tamoxifen-stimulated growth. It is therefore reasonable to speculate that surviving cells are selected to be exclusively oestrogen responsive but which do not possess the survival mechanisms to support tamoxifen-stimulated growth. Thus, tamoxifen would again express antitumour actions on oestradiol-stimulated tumour growth (Yao *et al*, 2000). Based on the laboratory findings (Yao *et al*, 2000), a clinical program should be initiated to determine the therapeutic value of short or extended conjugated equine oestrogen- or high phytoestrogen- containing diets. The results of the studies could establish new standards of clinical care initially to extend the effectiveness of antihormonal therapies for metastatic breast cancer and, subsequently, the consideration of rotating adjuvant antihormones with an ‘oestrogen purge’ to improve survival through extending the duration of disease-free survival.

The next two challenges have the potential to expand the value of the developing knowledge on ER-induced cellular apoptosis. Half of the ER-positive tumours do not respond to antihormonal therapy; therefore, any strategy to target the ER in endocrine refractory tumours would ultimately double response rates in breast cancer. Endocrine refractory disease appears to have developed survival pathways that are independent of regulation by ER. In otherwords, the pivotal role of the ER in growth has been subverted by alternate survival and growth pathways. One potential strategy could be to convert an endocrine nonresponsive tumour to become responsive to oestrogen-induced apoptosis by blocking the survival pathways with the expanding list of tyrosine kinase inhibitors and antibodies that block cell surface signalling. The goal would be to prevent cancer cell survival. This pharmacological strategy might enhance the possibility that the ER complex could initiate events which lead to apoptosis in the vulnerable cells. There would also be the possibility that the oestradiol ER complex could synergise with traditional inducers of apoptosis, for example, chemotherapy in cells paralysed with select cell survival inhibitors. All these options can be advanced in clinical trial.

The ultimate goal of the basic research will be to identify the molecular mechanism that permits the oestradiol ER complex to switch from being a survival mechanism through replication to a death signal. How does the ER complex know when it must induce the death of an aberrant cell? Clues are already available since the expression of ectopic ER in ER-negative cells prevents replication (Jiang and Jordan, 1992) so the next step will be to identify critical pathways in the ER-negative cell that can convert inhibition of replication to the induction of apoptosis.

## CONCLUSIONS

New knowledge about the ability of low concentrations of oestrogens to cause apoptosis in exhaustively treated breast tumour has the potential to advance targeted therapeutics not only in breast cancer but also in other cancers. The remarkable ability of the oestradiol ER complex, a natural signal-transduction pathway, to discriminate between a growth and a death environment is unique. Application of the new knowledge has immediate relevance for the treatment of metastatic breast cancer and the eventual maintenance of breast cancer patients for decades using cycles of antihormones, but with regular ‘oestrogen purges’ to kill resistant cells, and subsequently reactivate antihormonal therapy. Most importantly, the new knowledge will establish a strategic plan to integrate novel survival blockers into a logical treatment strategy and simultaneously utilise the emerging power of the ‘omics’ technologies to identify specific targets for future apoptotic therapy.
